# Nanopore-Based Surveillance of Zoonotic Bacterial Pathogens in Farm-Dwelling Peridomestic Rodents

**DOI:** 10.3390/pathogens10091183

**Published:** 2021-09-13

**Authors:** Nusrat A. Jahan, Laramie L. Lindsey, Evan J. Kipp, Adam Reinschmidt, Bradley J. Heins, Amy M. Runck, Peter A. Larsen

**Affiliations:** 1 Department of Veterinary and Biomedical Sciences, College of Veterinary Medicine, University of Minnesota, St. Paul, MN 55108, USA; jahan036@umn.edu (N.A.J.); linds758@umn.edu (L.L.L.); kipp0046@umn.edu (E.J.K.); adam.reinschmidt16@ncf.edu (A.R.); 2 Department of Animal Science, College of Food, Agricultural, and Natural Resource Sciences, University of Minnesota, St. Paul, MN 55108, USA; hein0106@umn.edu; 3 Department of Biology, Winona State University, Winona, MN 55987, USA; arunck@winona.edu

**Keywords:** agriculture, 16S amplicon sequencing, metabarcoding, nanopore sequencing, dairy cattle, One Health, *Peromyscus leucopus*, *Mus musculus*, *Blarina brevicauda*, *Rattus norvegicus*

## Abstract

The effective control of rodent populations on farms is crucial for food safety, as rodents are reservoirs and vectors for several zoonotic pathogens. Clear links have been identified between rodents and farm-level outbreaks of pathogens throughout Europe and Asia; however, comparatively little research has been devoted to studying the rodent–agricultural interface in the USA. Here, we address this knowledge gap by metabarcoding bacterial communities of rodent pests collected from Minnesota and Wisconsin food animal farms. We leveraged the Oxford Nanopore MinION sequencer to provide a rapid real-time survey of putative zoonotic foodborne pathogens, among others. Rodents were live trapped (n = 90) from three dairy and mixed animal farms. DNA extraction was performed on 63 rodent colons along with 2 shrew colons included as outgroups in the study. Full-length 16S amplicon sequencing was performed. Our farm-level rodent-metabarcoding data indicate the presence of multiple foodborne pathogens, including *Salmonella* spp., *Campylobacter* spp., *Staphylococcus aureus*, and *Clostridium* spp., along with many mastitis pathogens circulating within five rodent species (*Microtus pennsylvanicus, Mus musculus, Peromyscus leucopus, Peromyscus maniculatus,* and *Rattus norvegicus*) and a shrew (*Blarina brevicauda*). Interestingly, we observed a higher abundance of enteric pathogens (e.g., *Salmonella*) in shrew feces compared to the rodents analyzed in our study. Knowledge gained from our research efforts will directly inform and improve farm-level biosecurity efforts and public health interventions to reduce future outbreaks of foodborne and zoonotic disease.

## 1. Introduction

Rodents are the largest group of mammals in the world, and they are well known for harboring a plethora of zoonotic pathogens of concern for human and animal health [1]. Both native and invasive species of mice and rats benefit from human activities, especially agricultural systems. Rodents are a common hindrance of food production systems globally and they are known to transmit zoonotic pathogens to food animals and raw produce by contaminating the overall farm environment [2,3,4,5]. This transmission is largely due to the amplification of foodborne pathogens through the daily deposition of urine and fecal pellets into the production environment. For example, a single rodent within a barn or food-production facility can introduce upwards of 23 million *Salmonella* bacteria into production pipelines within 24 h [6,7]. However, the functional role that peridomestic (i.e., living in and around human habitations) rodents serve in the amplification and transmission of various zoonoses is likely underappreciated. Clear links have been identified between rodent pests and outbreaks of zoonotic diseases throughout Europe and Asia [8,9,10,11,12]; yet, little research has been devoted to studying this relationship in the United States [4,13]. Specifically, regional studies focused on specific rodent species and their pathogen reservoir status across the diverse agricultural landscapes of the United States are lacking. Hence, our overarching research goal was to investigate the role of rodent pests on food animal farms as reservoirs or carriers of zoonotic pathogens, especially with respect to species-specific patterns.

Emerging genomic technologies are providing exciting new opportunities for the surveillance of zoonotic pathogens in diverse settings and environments. Next-generation sequencing platforms allow for the metabarcoding of complex bacterial communities using taxonomically informative genes (e.g., the 16S rRNA gene). 16S rRNA sequence data are particularly useful as a molecular marker for bacterial identification, including for pathogens with clinical relevance [14,15]. The 16S rRNA gene has nine hypervariable regions (V1-V9) with varying levels of phylogenetic signal, of which the V3 and V4 regions are particularly useful for resolving genus and species-level relationships [16]. Second-generation sequencing platforms frequently used for bacterial metabarcoding experiments (e.g., Illumina MiSeq and NextSeq) provide high per-base accuracy and sequencing throughput; however, the resulting data consist of relatively short (~300 bp) reads, often permitting the analysis of particular subregions of the full-length (~1550 bp) 16S rRNA gene [17]. Alternatively, the Oxford Nanopore Technologies (ONT) MinION sequencer is a third-generation single-molecule sequencing platform that can sequence exceptionally long DNA fragments (i.e., thousands to millions of bases in length) [18,19]. For this reason, the MinION can sequence the entire ~1550 bp 16S rRNA gene, thus providing two to five times greater coverage of the 16S rRNA gene when compared to sequencing data originating from second-generation technologies. Full-length 16S sequence data provide a greater number of phylogenetically informative characters, thus enhancing downstream bacterial taxonomic assignment. This approach is important given that bacterial pathogenicity is typically considered a species or strain level phenomenon [20]. Although per-base accuracy of nanopore sequencing is lower (~98%) than that of more commonly used next-generation sequencing platforms (e.g., Illumina and Pacific Biosciences HiFi (i.e., circular consensus sequencing); both at >99.9% accuracy) similar or even greater taxonomic resolution is still achieved with the ONT MinION [21,22,23,24]. Furthermore, with continued technological and bioinformatic advancements, ONT-based DNA sequencing will continually improve over time [25,26].

With respect to metabarcoding, the ONT MinION platform has been successfully applied in several studies, including the characterization of bacterial mock communities [25,27,28]; microbiota profiling of species and tissues such as dog skin [29], canine feces [30], equine gut [31], water buffalo milk [32], sea louse [33], and microalgae [34]; identification of fungi [35]; and characterization of plastic-associated species in the Mediterranean sea [36]. Additionally, metagenetic analyses of environmental samples obtained from glacial regions [37], aquatic environments (e.g., ocean water column [38], river water [39], wastewater [40], and freshwater [41]), building dust [22], and the International Space Station [42] demonstrate the potential and applicability of nanopore sequencing for microorganism detection across diverse environments and field settings. Notably, nanopore sequencing has been used to describe human gut [43], and nasal microbiota [44], as well as those associated with colorectal cancer tumors [45], and thrombus samples [46]. Nanopore-based pathogen surveillance of EMS vehicles [15], prosthetic devices [47], hospitals [48], and antibiotic resistance markers in clinical samples [49,50,51,52] clearly demonstrate the potential of the MinION platform as a pathogen surveillance tool.

**Figure 1:**
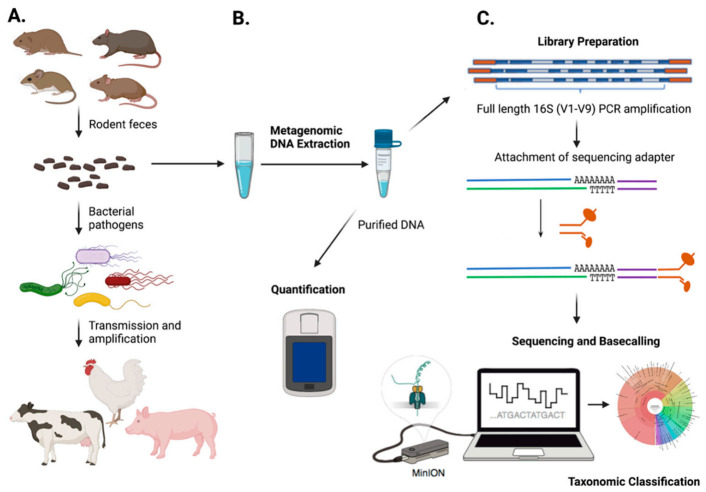
Workflow of the overall study design performed herein. (**A**): Rodents effectively serve as amplifiers of bacterial pathogens across a given farm environment through fecal deposition, including possible transmission to resident farm animals. (**B**): DNA extraction from rodent colon contents (i.e., feces) and quantification. (**C**): Laboratory workflow to monitor bacterial communities from rodent samples using nanopore sequencing.

In light of the growing number of studies showing the utility of the ONT MinION for a diverse range of biosurveillance applications and, given the lack of research on the rodent–agriculture interface in the USA, we set out to examine the potential of the MinION for metabarcoding rodent-borne zoonoses. Here, we show how the ONT MinION can be used to taxonomically characterize fecal bacterial communities of farm-dwelling rodents. Our study area included farms in the Upper Midwest of the United States (i.e., Minnesota and Wisconsin), regions where studies focused on the rodent–farm interface are lacking [1]. Our intent was twofold: (1) to elucidate the farm-level rodent diversity in our study area and (2) to use full-length 16S metabarcoding to identify rodent-borne zoonoses of agricultural concern (Figure 1).

## 2. Results

### 2.1. Rodent Trapping on Farms

We live-trapped 90 rodents during the course of our study; 29 from Farm A, 43 from Farm B and 18 from Farm C (Table 1). We additionally captured two shrews (*Blarina brevicauda*) on Farm C and included those in our analyses. We identified five rodent species across our study sites, including three native (*Peromyscus maniculatus, P. leucopus,* and *Microtus pennsylvanicus*) and two invasive species (*Mus musculus* and *Rattus norvegicus*). The captured shrew species, *B. brevicauda*, is native to the Midwest. The majority of rodent captures were centered around feed bunks, grain storage sites, and within cow barns.

### 2.2. Nanopore Sequencing Workflow of Full-Length 16S rRNA for Rodent Microbiome Analysis

We generated full-length 16S amplicon sequencing data comprising more than 33 million reads from 63 farm-caught rodent and 2 shrew colon extracts. Run 1 and Run 3 included 22 rodent colon samples from the large conventional Farm (A), Run 2 and Run 4 included 23 samples from the med-sized Farm (B), while Run 5 included all 20 samples from the small family Farm (C; Table 2). Each of the MinION sequencing runs included 12 molecularly barcoded samples except Run 5, which included 20 barcoded samples that were collected from individual rodent and shrew colons (Table 2). Average raw 16S rRNA reads generated across the five sequencing runs ranged from 3.5 to 9.4 million reads and mean quality scores of filtered reads ranged from 8.2 to 10.1 (Table 2). The per-base error rate of our MinION sequencing was approximately 1 in 11 bases, a result that is consistent with other nanopore studies [22,53]. However, read depths of full-length 16S amplicons averaged 16× coverage, resulting in high-quality consensus sequences and reducing concerns of per-base sequencing error [54]. To sort high-quality reads, pass fast5 reads were generated from raw data using the Guppy base calling program and FAST model setting. Although Run 3 data (~3.5 million reads) showed fewer sequencing reads than other sequencing runs, the mean Q score (8.2), mean read length (1625.40 bp) and read length N50 (1593 bp) were comparable (Table 2). For all sequencing runs, read lengths had a narrow distribution, with mean read lengths ranging from 1132.70 to 1625.40 bp, which was close to the full-length of the 16S rRNA gene (about 1550 bp). A filtering program (Cutadapt) was used to discard reads out of a 1200 to 1800 bp length range. Collectively, our quality control measures (see Methods) filtered out approximately 20% of initial raw reads from all runs.

### 2.3. Rodent Fecal Core Microbiome and Microbial Diversity

**Figure 2:**
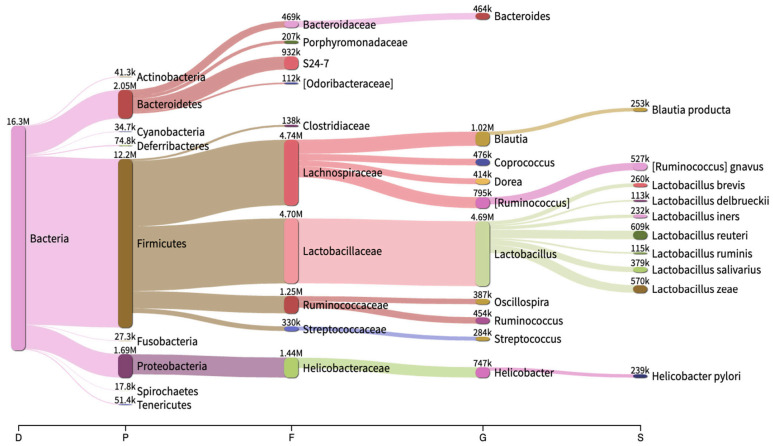
Core rodent fecal microbiome observed herein (*n* = 63). Numbers above nodes are reads assigned to each taxon.

After taxonomic classification, we obtained 96.25% of classified reads and 3.75% of unclassified reads. Total reads corresponding to bacteria were at 96.25%, and 0% reads were assigned to virus, fungi and protozoa. The microbial classifications were obtained at different taxonomic levels (e.g., division, phylum, class, order, family, genus, and species) for all of the 65 colon extract samples. Overall, the most abundant phylum for all five rodent species was Firmicutes (~75% of total reads), followed in abundance by Bacteroidetes (12.5%) and Proteobacteria (~10%), and other phyla comprised less than ~2.5% of total classified phyla (*n* = 80; Figure 2).

At the rodent species level, the house mouse (*Mus musculus*) was the most captured species, with a total of 48 animals trapped from Farms A and B. After taxonomic classification of the combined house mouse reads, 77 phyla, 886 genera, and 473 bacterial species were obtained. At the genus and species levels, a filtering step was applied that included a threshold of 100 reads binned to the respective taxonomic level, which retained 261 genera and 181 species passing the threshold. For *M. musculus*, the most abundant genera were *Lactobacillus* (27.7%), *Ruminococcus* (10%), *Helicobacter* (8%), *Bacteroides* (7.6%), and *Blautia* (6.8%) (see Supplementary Table S1). Other abundant genera included fecal or mammalian gut microbiota (e.g., *Coprococcus, Faecalibacterium, Dorea, Roseburia, Oscillospira*) and potential human pathogens and bovine mastitis-causing pathogens such as *Staphylococcus, Streptococcus, Bacillus,* and *Enterococcus*, each constituting more than 1% of the total classified reads.

The second most dominant rodents in across our study area consisted of *Peromyscus* spp., with a total of 29 captured from Farms A, B, and C. After combining and taxonomically classifying all *Peromyscus* 16S rRNA reads, 58 phyla, 619 genera, and 344 species were obtained. After threshold filtering, 139 genera and 89 species were retained. The most abundant genus was *Lactobacillus* (37.6%), followed in abundance by *Ruminococcus* (16.5%), *Blautia* (10.6%), *Dorea* (4.6%), *Helicobacter* (4%), and *Streptococcus* (3.4%). Other fecal-related genera formed less than ~3% of the total (see Supplementary Table S1).

Brown rats (*Rattus norvegicus*) were captured from Farms A and C with a total of seven animals. The combined nanopore reads were taxonomically classified into 47 phyla, 583 genera and 357 species. Threshold filtering retained 119 genera and 88 species, where *Lactobacillus* (22%), *Blautia* (17.3%), *Ruminococcus* (11.5%), *Streptococcus* (7.8%), and *Dorea* (6.5%) were the most abundant genera (see Supplementary Table S1). Other mammalian gut microbiota (e.g., *Oscillospira, Coprococcus, Faecalibacterium, Roseburia*) and potential human pathogenic genera (*Helicobacter, Prevotella, Staphylococcus, Clostridium, Bacteroides*) compiled more than 23% of the bacterial genera.

We captured a total of six meadow voles (*Microtus pennsylvanicus*) from Farms B and C. Taxonomic classification of vole 16S rRNA reads revealed 52 phyla, 542 genera and 302 species. Subsequent filtering retained 121 genera and 61 species, with the most prominent genera in the meadow vole feces consisting of *Lactobacillus* (24%), *Ruminococcus* (19.6%), *Blautia* (11.7%), *Oscillospira* (7.6%), and *Coprococcus* (5.3%) (see Supplementary Table S1).

**Figure 3:**
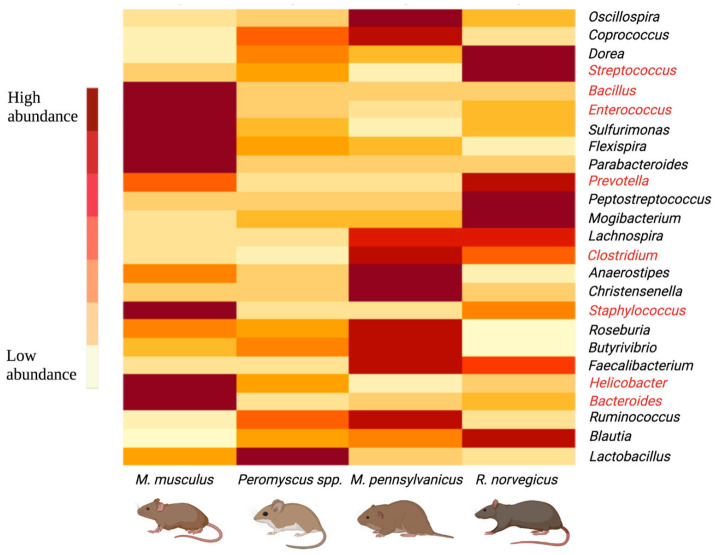
Heatmap of the most abundant (>1%) bacterial genera, across rodent species, identified by mapping 16S rRNA gene amplicons against the GreenGenes reference database. Genera having low relative abundance are light in color, while those with high abundance are dark. Genera that are pathogenic to humans appear in red font.

While examining species level composition, several bacterial species were abundant and observed across all five rodent species (Figure 3). For example, abundant *Lactobacillus* species included *L. reuteri, L. zeae, L. salivarius, L. delbrueckii, L. brevis, L. helveticus, L. ruminis,* and *L. iners*. Another dominant genus *Ruminococcus* consisted of *R. gnavus, R. torques, R. flavefaciens, R. bromii,* and *R. callidus*. *Blautia* species included *B. producta* and *B. obeum*. The following species were dominant across all rodent samples in our study: *Dorea formicigenerans, Roseburia faecis, Prevotella copri, Faecalibacterium prausnitzii, Oscillospira guilliermondii, Clostridium perfringens, Helicobacter pylori,* and *Coprococcus eutactus*. The most abundant *Staphylococcus* species included *S. aureus, S. epidermidis, S. haemolyticus,* and *S. sciuri*. The most abundant *Streptococcus* species included *S. luteciae, S. anginosus, S. alactolyticus,* and *S. infantis.*

### 2.4. Shrew Fecal Core Microbiome and Microbial Diversity

**Figure 4:**
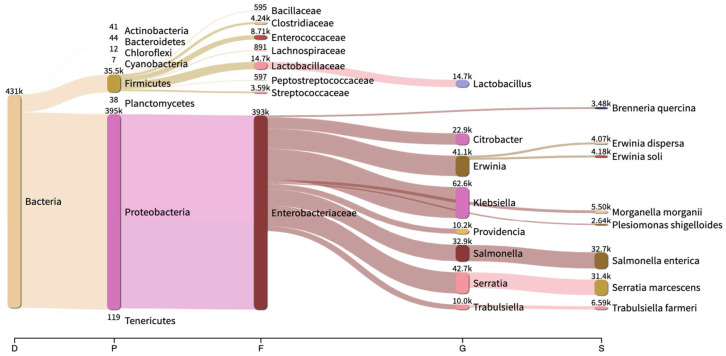
Shrew (*B. brevicauda*) fecal microbiome observed herein (*n* = 2). Numbers above nodes are reads assigned to each taxon.

Two shrews from the same species, *Blarina brevicauda,* were captured from a small family farm (Farm C). Taxonomic classification analysis of the shrew 16S sequencing data revealed 28 phyla, 281 genera, and 178 species. The most abundant phylum for both shrews was Proteobacteria (~91% of total reads), followed in abundance by Firmicutes (8%), and other phyla comprised less than ~1% of total classified phyla (*n* = 28; Figure 4).

**Figure 5:**
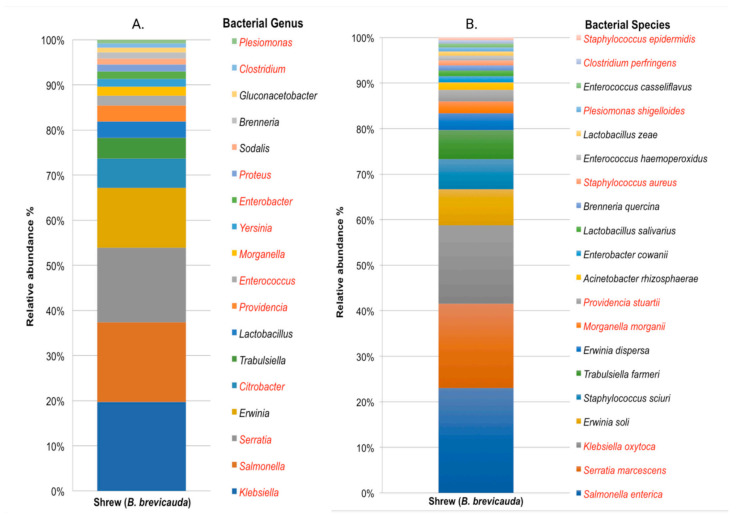
Shrew (*B. brevicauda*) microbiota representing > 1% relative abundance at genus (**A**) and species (**B**) levels. Potential pathogenic genera (**A**) and species (**B**) to humans appear in red font.

At the genus level, a total of 281 genera were identified and 36 genera were retained after applying a threshold of 100 reads binned to each genus. The shrew fecal microbiome was rich in *Klebsiella* (18.8%), followed in abundance by *Salmonella* (16.8%), *Serratia* (15.7%), *Erwinia* (12.6%), and *Citrobacter* (6.2%) (see Supplementary Table S1). Moreover, it contained other fecal-related and potential pathogenic genera, including *Providencia, Enterococcus, Morganella, Yersinia, Enterobacter, Proteus, Clostridium, Plesiomonas, Vibrio, Bacillus, Pseudomonas, Staphylococcus,* and *Streptococcus*, representing less than 5% each of the total bacterial composition. The relative abundance of the 18 most abundant taxa determined at the genus level is shown using bar graphs in Figure 5.

Metabarcoding analyses at the species level revealed a total of 178 species, with 29 retained after applying a threshold of 100 reads binned to each species. *Salmonella enterica* was the most abundant species with 21.5% reads assigned, followed in abundance by *Serratia marcescens* (17.3%), *Klebsiella oxytoca* (16%), *Erwinia soli* (7.4%), *Staphylococcus sciuri* (6.2%), and *Trabulsiella farmeri* (6%). Other abundant species included putative human and plant pathogens such as *Morganella morganii, Providencia stuartii, Enterobacter cowanii, Staphylococcus aureus,* and *Brenneria quercina*, each >1% of the total bacterial species composition.

## 3. Discussion

We used MinION nanopore sequencing to metabarcode fecal microbial communities in peridomestic small mammals (i.e., rodents, shrews). Rodents and shrews within our study were collected from three dairy and mixed animal farms over a two-year period. Our nanopore-based metabarcoding pipeline (Figure 1) demonstrates the utility of the MinION technology for the surveillance of pathogenic organisms in peridomestic pests [55,56,57,58,59] and, when used for farm-level surveillance, the methodology can be leveraged to inform and strengthen biosecurity practices. The long read length, depth of coverage, and rapid results make MinION sequencing a robust option for pathogen surveillance. Although nanopore sequencing has a higher per-base accuracy error rate than other next-generation sequencing methods [53], we based our taxonomic classification on read depths of over 30 million full-length 16S (~1.5 kb) reads. We acknowledge that a high number of sequencing reads for putative pathogens does not necessarily indicate the absolute presence of the organism; thus, standard culturing techniques and related molecular methods are still valuable tools for the confirmation of putative pathogens. Nevertheless, for our purpose of a primary screening tool, MinION 16S sequencing was a cost effective and rapid method that achieved our surveillance goals. Regarding the usage of 16S rRNA gene sequence data for the taxonomic classification of bacteria, we note that shotgun metagenomic approaches (e.g., deep sequencing DNA isolates with second-generation Illumina technologies) can provide a more accurate depiction of the diversity of a given microbial community than single-gene sequence data. Moreover, such approaches can also recover detailed profiling of antimicrobial-resistant genes within a sample. Despite these observations, there are clear benefits with respect to MinION 16S rRNA sequencing, as the method can be performed within individual labs (or in the field for real-time surveillance) and can effectively identify bacteria that are abundant within a given sample.

To the best of our knowledge, this is the first study to reveal and compare the composition of bacterial communities in gastrointestinal tracts of wild-caught rodents and shrews (e.g., *M. musculus, Peromyscus spp., M. pennsylvanicus, R. norvegicus* and *B. brevicauda*) using a third-generation sequencing technology. Rodents are considered to be among the most commensal synanthropic animals, especially within the context of food-production systems. Contrastingly, shrews (*B. brevicauda*) naturally live in woodlands, cultivated fields, vegetable gardens and mainly feed on invertebrates [60,61]. Both rodents and shrews retreat into barns, cellars and sheds during fall and winter months (both for shelter and foraging), thus providing opportunities for direct and indirect (i.e., with rodent/shrew feces and urine) contacts with humans, pets, livestock, and poultry. Such interactions have the potential for the transmission of rodent-borne zoonoses, especially pathogenic bacteria [1,62].

For all five rodent species, phylum level gut microbiomes were similar to that of other rodent gut microbiomes reported in the scientific literature, with Firmicutes, Bacteroidetes and Proteobacteria comprising more than 97% of the gut microbiota [63,64]. Firmicutes was the most abundant phylum in all rodent species, ranging from 64% to 91.5%. In shrews, the most abundant phylum was Proteobacteria, covering 91.7% of the total shrew microbiota. At the genus level, *Lactobacillus* was the top genus for all the rodent species in our study, which is in line with the findings of a previously reported study on laboratory rodent microbiomes (see Supplementary Table S1) [65]. This finding implies that the core fecal bacterial compositions of wild and laboratory rodents share a degree of similarity. *Lactobacillus* also constitutes a significant component of the human gut microbiome [66]. Furthermore, *Ruminococcus* was the second most prominent genus in all mouse species (*M. musculus, Peromyscus* spp., *M. pennsylvanicus*), whereas *Blautia* was prominent in rats (*R. norvegicus*). Alternatively, *Klebsiella* was the most prominent genus in the northern short-tailed shrew feces, followed by *Salmonella, Serratia* and *Erwinia*. Of note is that metabarcoding data are scant across all shrews, thus limiting genus or species-level comparisons. A single study originating from China included the Asian house shrew (*Suncus murinus*) in their 16S rRNA metabarcoding analyses and identified *Clostridium* as the most abundant genus in their sample [67].

**Figure 6:**
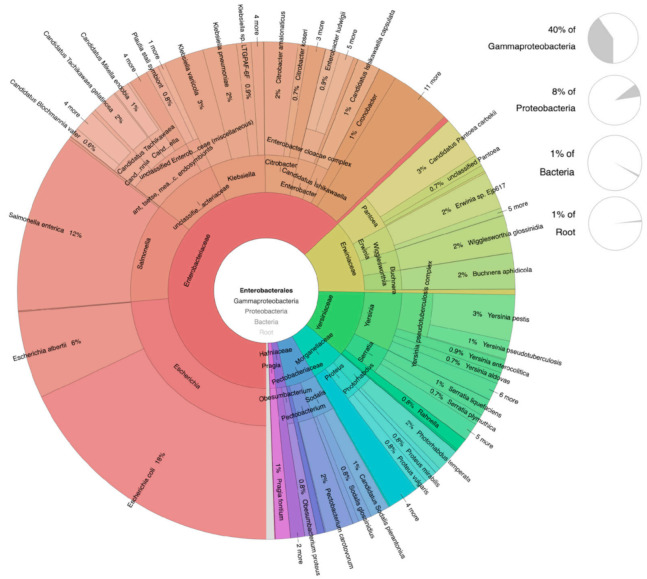
Krona plot showing overall rodent and shrew-associated (*n* = 65) bacterial species abundance within the order Enterobacteriales.

We identified a higher relative abundance of potential human and animal pathogens in shrew fecal samples than in rodent fecal samples. In brief, the resulting data indicate the presence of multiple putative foodborne pathogens from the Enterobacteriales order, including *Salmonella* spp., *Shigella* spp., *Plesiomonas shigelloides, Yersinia* spp., and *Escherichia coli*, in all small mammal species (Figure 6). Other top foodborne pathogens observed in our analysis included *Listeria monocytogenes*, *Campylobacter* spp., *Clostridium perfringens, Vibrio* spp., and *Staphylococcus aureus*. These are identified as major bacterial pathogens that cause foodborne illness and hospitalization in the USA and globally each year [68].

**Figure 7:**
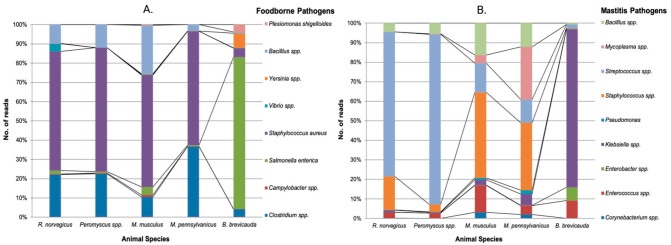
Abundance of foodborne (**A**) and mastitis (**B**) pathogens in all small mammal species.

Interestingly, *Salmonella enterica* was the most abundant species (~21.5%) in the shrew feces but was much lower across our rodent samples (Figure 7). On the contrary, *Clostridium perfringens, Bacillus* spp., and *Staphylococcus aureus* were abundant within rodents when compared to the two shrew samples. Within our rodent sample, *Vibrio* spp. and *Campylobacter* spp. were observed in comparatively higher abundance in *R. norvegicus* and *M. musculus*, respectively. These bacteria can be transmitted by contaminated food and water, and, within our study, they had a greater relative abundance in shrew fecal samples than in those of rodents.

Mastitis is a leading cause of cow culling and causes substantial economic losses to the dairy industry [69]. Because a majority of our rodent samples were collected from large-sized (Farm A; ~20,000 cattle) and medium-sized (Farm B; ~600 cattle) dairy farms, we investigated the presence and abundance of pathogens causing bovine mastitis in the fecal samples of the farm-dwelling rodents. Many important mastitis-causing pathogens, including *Streptococcus* spp., *Staphylococcus* spp., *Klebsiella* spp., *Enterococcus* spp., *Enterobacter* spp., *Mycoplasma* spp., and *Corynebacterium* spp., were observed in varying abundance across our rodent and shrew samples. *E. coli, Streptococcus* spp., *Staphylococcus* spp., and *Corynebacterium* spp. are well known environmental mastitis pathogens [70], and the presence of these pathogens in the resident rodent population of each dairy farm is a putative health risk to the resident cattle, especially when considering the fecal output of commensal rodent species into the farm environment [1]. Our metabarcoding data indicate that the rodents sampled during our trapping events are possible reservoirs of mastitis pathogens and have the potential to continuously introduce these pathogens into the dairy farm environments. Moreover, they can amplify and mechanically vector these pathogens from sick to susceptible animals through fecal-based pathogen amplification [1]. When performing rodent species comparisons of the relative abundance of mastitis pathogens across Farms A, B, and C (Figure 7B), the house mouse (*M. musculus*) exhibited the highest number of mastitis-associated pathogens. Given this observation and in light of our farm-level observations, we hypothesize that because *M. musculus* cohabitates with cows inside barns (i.e., tunneling and nesting extensively in bedding material and wall insulation), they are exposed to a greater overall amount of mastitis pathogens by direct and indirect interactions with resident dairy cow herds.

Rodent and shrew-borne bacterial pathogens can cause human diseases through various routes, especially by urine and fecal output into water and food resources. In our study, rodent fecal samples contained a variety of zoonoses of concern to human health in high abundance, including: *Helicobacter pylori*, known to cause chronic gastritis, gastric ulcers, and stomach cancer [71]; *Prevotella copri*, associated with the pathogenesis of rheumatoid arthritis [72]; pathogens related to nosocomial infections such as *Morganella morganii, Serratia marcescens,* and *Providencia stuartii* [73,74]; and opportunistic pathogens associated with splenic abscess (*Parabacteroides distasonis*) and anaerobic peritoneal infections (*Bacteroides fragilis*) [75,76].

Rodents are well-known across the globe for their commensal nature, living in close proximity to populations of both humans and domesticated species. An increasing number of studies suggest that rodents may serve as potential sources of infectious zoonotic diseases via pathogen amplification and cross-species transmission [1]. Surprisingly little attention has been paid to rodents associated with food-production systems in the United States, despite the fact that they occupy food animal farms, fresh produce lands, and processing facilities across the country. In our study, a substantial number of sequences obtained from the northern short-tailed shrew were identified as potential foodborne pathogens. It is possible that *B. brevicauda* is a reservoir of bacterial foodborne pathogens; however, shrews are not considered peridomestic species and thus transmission risk is likely quite low. Alternatively, invasive *M. musculus* and *R. norvegicus* and native *Peromyscus* spp. readily adapt to farm environments and have the capacity to establish abundant on-farm populations. We recorded evidence of large on-farm populations of both *M. musculus* and *R. norvegicus* consisting of subfloor tunneling in barns and outbuildings, nesting within wall insulation and livestock bedding, and accessing livestock food storage areas. Despite observing a large population of *R. norvegicus* on Farm A, our live-trapping methods were not ideal for collecting *R. norvegicus.* Enhanced species-specific trapping efforts with pre-baiting measures to acclimate the rats and the use of multiple trapping methods might overcome trap avoidance behaviors exhibited by *R. norvegicus* [77]. Our personal observations during farm-level trapping and subsequent bacterial metabarcoding data indicate that farm-dwelling rodents are potential reservoirs of many putative zoonotic foodborne and mastitis-associated pathogens. In light of these results, we strongly recommend additional research focused on the rodent–agricultural interface across the United States.

## 4. Materials and Methods

### 4.1. Rodent Trapping and Sample Collection on Farms

During the summer (2019) and fall (2019, 2020), we collected rodents from one dairy cattle farm (A), one mixed animal (dairy cattle and hog) farm (B) and another mixed animal (cattle and horse) farm (C). Farms A and B are located in Nicollet and Stevens counties of Minnesota, respectively, and Farm C is in southeastern Wisconsin in Sauk County. Farm A is a large dairy operation (~20,000 dairy cattle), Farm B is a medium-sized operation (600 dairy cattle and 400 hogs), and Farm C is a small-sized family farm (cattle and horses < 100 animals). Rodent activity was elevated on farms per observations by farm managers, particularly on Farm A where we observed hundreds of Norway rats (*R. norvegicus*) actively foraging around compost piles during daylight hours. All farms had poison bait stations, kill traps and cats as rodent control measures during the time of our visits. Four nights of rodent trapping were conducted at each study site using 150 Sherman live traps baited with oats. Decontamination of all traps was performed using a 10% sodium hypochlorite solution (10 min soak) before and after each trapping event. All trapped animals were humanely euthanized following approved UMN IACUC protocols (protocol number 1809-36374A). Small mammals were collected with approval by the Minnesota Department of Natural Resources under Special Permit No. 23896. Two shrews (*Blarina brevicauda*) were collected during the course of our research and were included in our analyses. Standard morphological techniques were used to identify rodents and shrews to species-level and metadata (e.g., species, age, weight, sex, body measurements) were collected for each individual animal. Biological samples (e.g., feces, colon) were collected and preserved (e.g., liquid nitrogen, freezer) for metabarcoding analysis and further quantification of pathogens of interest. A schematic workflow of the overall study design is shown in Figure 1 and specimens examined are provided in Table S2.

### 4.2. DNA Extraction

DNA was extracted with a QIAamp PowerFecal Pro DNA Kit (QIAGEN, Hilden, Germany). Snap-frozen rodent and shrew feces and colon extracts were stored at −80 °C and were used for DNA extraction. Briefly, 250 mg of colon contents were added to PowerBead Pro tubes and 800 μL of solution CD1 was mixed by vortexing. A bench top PowerLyzer 24 Homogenizer (QIAGEN, Hilden, Germany) was used for homogenizing the samples at 2000 rpm for 30 s, pausing for 30 s, then homogenizing again at 2000 rpm for 30 s to enhance cell lysis. PowerBead Pro tubes were centrifuged at 15,000× *g* for 1 min, and the resulting supernatant was transferred to clean microcentrifuge tubes. We used fully automated QIAcube connect instruments (QIAGEN, Hilden, Germany) for DNA extraction following the manufacturer’s instructions. DNA concentrations were measured by fluorescence in a Qubit 4 fluorometer (Thermofisher Scientific, Waltham, MA, USA) using the Qubit dsDNA BR Assay Kit (Thermofisher Scientific, Waltham, MA, USA) following the manufacturer’s instructions.

### 4.3. Nanopore Library Construction and Sequencing

The 16S Barcoding Kit (SQK-RAB204; Oxford Nanopore Technologies, Oxford, UK) was used to prepare the amplicon library, following the manufacturer’s instructions for 1D sequencing strategy. The 16S region (1.5 kb) of bacteria was amplified using specific primers (27F-1492R) and subsequently barcoded. This approach enables targeted sequencing of multiple samples and provides genus-level resolution. Five sequencing runs were performed with a total of 65 samples, including 11 (run 1, 3, 4), 12 (run 2), and 20 (run 5) barcoded samples from individual rodents and shrews. Briefly, genomic DNA samples were diluted to 100 ng/μL and amplification of the full-16S rRNA gene was performed by PCR with reaction volume of 50 μL, using the primers 27F 5′-AGAGTTTGATCCTGGCTCAG-3′ and 1492R 5′-GGTTACCTTGTTACGACTT-3′, and Taq DNA polymerase LongAmp (NewEngland Biolabs, Ipswich, MA, USA). Amplification was performed using Bio-Rad Laboratories PCR Thermal Cycler T100™ (Bio-Rad Laboratories, Hercules, CA, USA) with the following PCR conditions: initial denaturation at 95 °C for 1 min, 25 cycles of 95 °C for 20 s, 55 °C for 30 s, and 65 °C for 2 min, followed by a final extension at 65 °C for 5 min.

PCR products (50 μL each) were purified with 30 μL Agencourt AMPure XP beads and incubated in a HulaMixer for 5 min at room temperature. After a magnetic bead washing step, purified products were eluted in 10 μL of elution buffer (10 mM Tris-HCl pH8.0 with 50 mM NaCl). The amount and purity of the sequencing library was quantified using a Qubit 4 fluorometer (ThermoFisher Scientific, Waltham, MA, USA) following the manufacturer’s instructions. Libraries were pooled in multiplex mode following the addition of 1 μL of rapid adapter (Oxford Nanopore Technologies, Oxford, UK) and incubated at room temperature for 5 min. The amplicon library (11 μL) was then diluted with a running buffer (35 μL) containing 3.5 μL of nuclease-free water and 25.5 μL of loading beads. Five nanopore sequencing libraries were separately run on FLO-MIN106 R9.4 (run 1, 2, 4, 5) and FLO-MIN111 R10.3 (run 3) flow cells (Oxford Nanopore Technologies, Oxford, UK). Sequencing runs were performed for 48 h. using the MinION control software, MinKNOW 4.0.20 (Oxford Nanopore Technologies, Oxford, UK).

### 4.4. Bioinformatic Analyses

After the completion of each sequencing run, raw signals in nanopore fast5 files were base-called using Guppy (version3.2.2, Oxford Nanopore Technologies, Oxford, UK), and a quality filter step was applied to retain only sequences with a mean Q-score ≥ 7. De-multiplexing of the barcoded samples was conducted using Porechop [78]. Adapter trimming and a second round of de-multiplexing were performed using Cutadapt 1.91 [79]. Only reads between 1200 and 1800 bp were selected for further analysis using Cutadapt. Read statistics for each sequencing run were obtained using Nanostat and NanoPlot [80]. For taxonomic assignments, Kraken2 [81] and Bracken [82] were used with the Greengenes (GG) database (https://benlangmead.github.io/aws-indexes/k2, accessed on 26 March 2021). While generating the Bracken classification report, a threshold of >100 reads was applied for higher confidence at the genus and species levels. For visualization Krona tools and Pavian interactive applications were used to generate taxonomic charts and flow diagrams [83,84]. The ggplot2 package (version 3.2.1) in RStudio software (version 3.3.3) was used to create a heatmap [85]. BioRender was used for illustrations and diagrams (Created with BioRender.com). Base-called data were uploaded to the EPI2ME interface, a platform for cloud-based analysis of MinION data, and WIMP (ONT; “What’s in my pot” software) analysis was performed in parallel to compare results.

## Figures and Tables

**Table 1:** Index of all captured animals from Farm A, B and C.

Rodent Species	Farm A	Farm B	Farm C
House mouse (*Mus musculus*)	27	21	0
White-footed mouse (*Peromyscus leucopus*)	0	13	11
Deer mouse (*Peromyscus maniculatus*)	1	4	0
Meadow vole (*Microtus pennsylvanicus*)	0	5	1
Norway rat (*Rattus norvegicus*)	1	0	6
**Shrew Species**			
Northern short-tailed shrew (*Blarina brevicauda*)	0	0	2
Total = 92	29	43	20

**Table 2:** Summary statistics of 16S nanopore sequencing of rodent colon contents performed herein. N = number of rodent samples barcoded and pooled on each MinION sequencing experiment. BP = Base Pairs.

MinION Sequencing Run	Farm (N)	Active Pores avg.	Read Count (Unit Million Reads)	Mean Read Length	Mean Q Score	Read Length N50	Total bp	QC > Q7
1	A (12)	509	5.0	1132.70	9.3	1564	5.7 × 10^9^	85.80%
2	B (11)	500	4.5	1178.30	8.3	1587	5.3 × 10^9^	85.50%
3	A (11)	509	3.5	1625.40	8.2	1593	5.7 × 10^9^	68.50%
4	B (11)	500	9.4	1477.10	10.1	1576	13.9 × 10^9^	85.40%
5	C (20)	504	5.8	1472.30	8.4	1564	8.6 × 10^9^	77.70%

## Data Availability

All nanopore sequence data are available on the National Center for Biotechnology Information Sequence Read Archive website under project accession number PRJNA759117.

## Supplementary Materials

The following are available online at https://www.mdpi.com/article/10.3390/pathogens10091183/s1, Table S1: Percentages of full-length 16S rRNA reads taxonomically assigned to bacterial genera per rodent and shrew species. Listed genera represent the top 15 of each rodent and shrew species, collectively; Table S2: specimens examined.
